# Rehabilitation Strategy Guided by Physiological Indicators in a Patient With Tetanus and Multiple Fractures: A Case Report

**DOI:** 10.7759/cureus.110348

**Published:** 2026-06-06

**Authors:** Tatsurou Kon, Akinori Matsumoto, Yuichiro Sasamoto

**Affiliations:** 1 Physical Medicine and Rehabilitation, Ohta General Hospital Foundation, Ohta Nishinouchi Hospital, Koriyama, JPN; 2 Emergency Medicine, Tsuchiya General Hospital, Koriyama, JPN; 3 Physical Therapy, School of Health Sciences, Fukushima Medical University, Fukushima, JPN

**Keywords:** autonomic instability, fractures, japanese rehabilitation, post-intensive care syndrome, tetanus

## Abstract

Tetanus is a serious infectious disease characterized by muscle rigidity and autonomic storm. In severe cases, long-term deep sedation is required, resulting in a high risk of post-intensive care syndrome (PICS). However, criteria for determining the timing of early rehabilitation intervention have not yet been established. We report the case of a woman in her 70s who developed tetanus following a wood-splinter injury to her right forearm and subsequently suffered bilateral humeral fractures due to muscle rigidity and convulsions. She was admitted to the intensive care unit (ICU) with an Ablett classification of Grade III, which progressed to Grade IV following ICU admission. On Day 11, rehabilitation was initiated after confirming the resolution of muscle hypertonicity and opisthotonos, a downward trend in creatine kinase (CK) levels, and the stabilization of hemodynamic parameters. No apparent recurrence of autonomic symptoms was observed during rehabilitation. The patient was gradually mobilized from edge-of-bed sitting to wheelchair use, and then to standing and walking. With multidisciplinary collaboration, she was discharged home on Day 114. The findings from this case may provide a basis for determining the timing of safe rehabilitation intervention in patients with severe tetanus.

## Introduction

Tetanus is caused by a neurotoxin from *Clostridium tetani* that inhibits inhibitory interneurons in the central nervous system, resulting in muscle rigidity and convulsions [1]. In severe cases, particularly those classified as Grade III-IV on the Ablett scale, patients may experience fractures due to muscle rigidity, as well as autonomic storm, including life-threatening arrhythmias and severe blood pressure fluctuations, and respiratory complications such as laryngospasm and aspiration pneumonia [2]. Therefore, strict systemic management in the intensive care unit (ICU), including isolation from external stimuli and the use of sedatives and muscle relaxants, is necessary [3]. However, these treatment measures are associated with a high risk of developing post-intensive care syndrome (PICS) [4]. While early rehabilitation intervention has been reported to be potentially beneficial for preventing PICS [5], clear intervention guidelines regarding timing and intensity have not yet been established [6]. In this report, we present a case in which a patient with bilateral humeral fractures complicated by tetanus underwent a stepwise rehabilitation program, guided by physiological indicators (including muscle tone, opisthotonos, creatine kinase (CK) levels, blood pressure, and heart rate) as supplementary criteria for determining the timing of rehabilitation intervention, resulting in successful discharge home.

## Case presentation

Table 1 shows the patient's clinical course following admission, and Table 2 shows the course of rehabilitation.

**Table 1:** Clinical course following admission CK: Creatine kinase; CRP: C-reactive protein; ICU: Intensive care unit; WBC: White blood cell count; -: Not measured or not applicable.

Day	Clinical events	Ablett grade	WBC (×10³/μL)	CRP (mg/dL)	CK (U/L)	Autonomic symptoms
Day 1	ICU admission intubation	IV	174	1.66	616	Present: autonomic storm and opisthotonos
Day 2	Autonomic storm with rapid vital sign fluctuations	IV	118	6.07	1107	Present: rapid vital sign fluctuations
Day 10	CK peak	IV	144	4.75	2013	Present
Day 11	CK declining hemodynamic stabilization	IV	97	4.32	1852	Resolved: muscle hypertonicity and opisthotonos absent
Day 22	Left humeral fracture identified	IV	82	9.46	149	No recurrence
Day 25	Tracheostomy	IV	56	5.26	65	No recurrence
Day 35	Edge-of-bed sitting initiated; no autonomic symptoms recurred	-	63	0.9	41	No recurrence
Day 41	Mechanical ventilation discontinued	-	76	1.93	28	No recurrence
Day 42	-	-	97	1.07	22	No recurrence
Day 53	-	-	69	0.26	18	No recurrence
Day 70	-	-	107	1.5	15	No recurrence
Day 86	-	-	69	0.65	17	No recurrence
Day 114	Discharged home	-	50	<0.05	47	No recurrence throughout

**Table 2:** Rehabilitation progression and functional assessment Due to bilateral upper limb fractures, MRC assessment was limited to the lower limbs (maximum possible score: 30 out of 60). RASS scores are reported from Day 19 onward, when formal sedation monitoring was initiated following a reduction in sedative dosage. The RASS is used with permission: © 2012 Virginia Commonwealth University, Richmond, VA, all rights reserved. The CAM-ICU is used with permission: © 2002, E. Wesley Ely, MD, MPH, and Vanderbilt University, Nashville, TN, all rights reserved. BI: Barthel Index; CAM-ICU: Confusion Assessment Method for the ICU; MRC: Medical Research Council scale; NA: Not assessable; PROM: Passive range-of-motion exercises; PT: Physical therapy; RASS: Richmond Agitation-Sedation Scale.

Day	Phase	Rehabilitation	RASS	MRC score	CAM-ICU	BI
Days 1–10	Pre-rehabilitation	None	NA	NA	NA	NA
Day 11	Rehab initiation (Days 11–41)	PROM and respiratory PT initiated	NA	NA	NA	0
Day 13	Rehab initiation	Postural drainage and respiratory PT	NA	NA	NA	0
Day 35	Rehab initiation	Edge-of-bed sitting initiated (3 min)	-1	NA	Positive	0
Day 41	Rehab initiation	Mechanical ventilation discontinued	-1	NA	Positive	0
Day 42	Mobilization (Days 42–69)	Wheelchair transfers initiated	+1	12	Positive	5
Day 53	Mobilization	Standing exercises initiated	+1	12	Positive	15
Day 70	Gait training (Days 70–114)	Gait training initiated	0	12	Negative	15
Day 83	Gait training	Multidisciplinary conference; home discharge goal set	0	18	Negative	40
Day 86	Gait training	Walking with a quad cane (minimal assistance)	0	18	Negative	50
Day 88	Gait training	Walking distance increased to 50 m	0	18	Negative	60
Day 92	Gait training	Step negotiation exercises (home environment simulation)	0	18	Negative	70
Day 114	Discharge	Discharged home; ambulation at supervision level	0	18	Negative	55

History, admission, and initial management (days 1-10)

**Figure 1:**
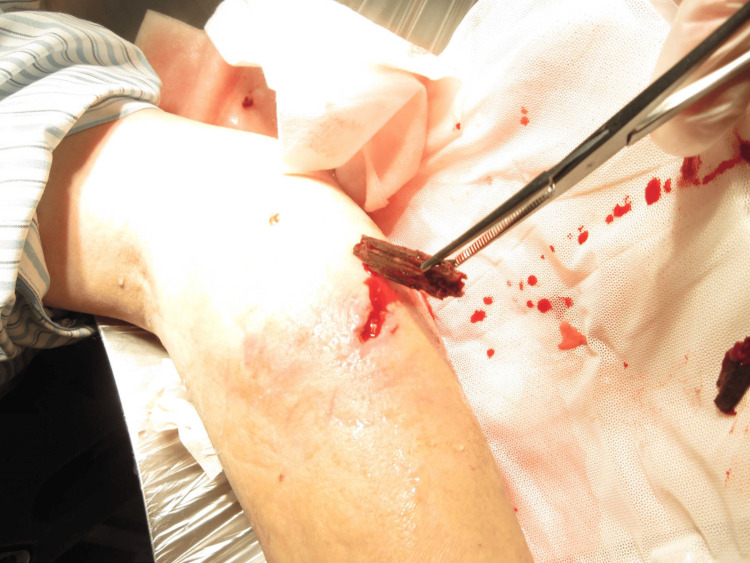
Wood fragment foreign body removed from the right upper limb A wood fragment foreign body was extracted from the right upper limb. The wound appearance following removal is presented in this image.

The patient was a woman in her 70s who worked as a housewife, with a height of 1.57 m, a weight of 40.8 kg, and a body mass index (BMI) of 16.6 kg/m². Her past medical history was significant for hypertension, for which she was receiving amlodipine and imidapril. Before admission, she was independent in activities of daily living (ADL), with a Barthel Index (BI) of 100 points [7]. She lived with her husband, while other family members resided far away. On Day -7, she sustained an injury to her right forearm when a wood splinter penetrated the skin (Figure 1).

**Figure 2:**
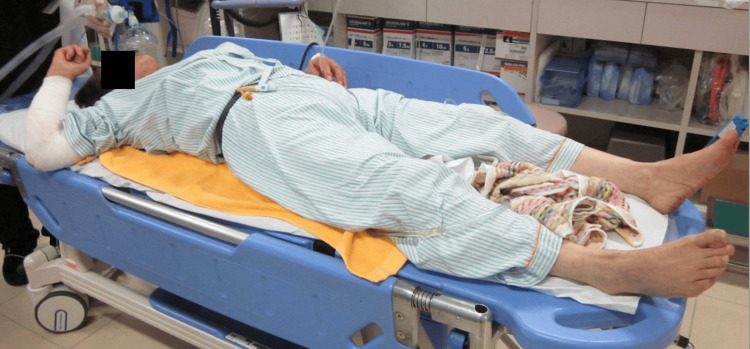
General physical examination in the emergency department Excessive trunk extension due to opisthotonos and muscle hypertonicity in the limbs is observed. These findings were consistent with a clinical diagnosis of tetanus.

**Figure 3:**
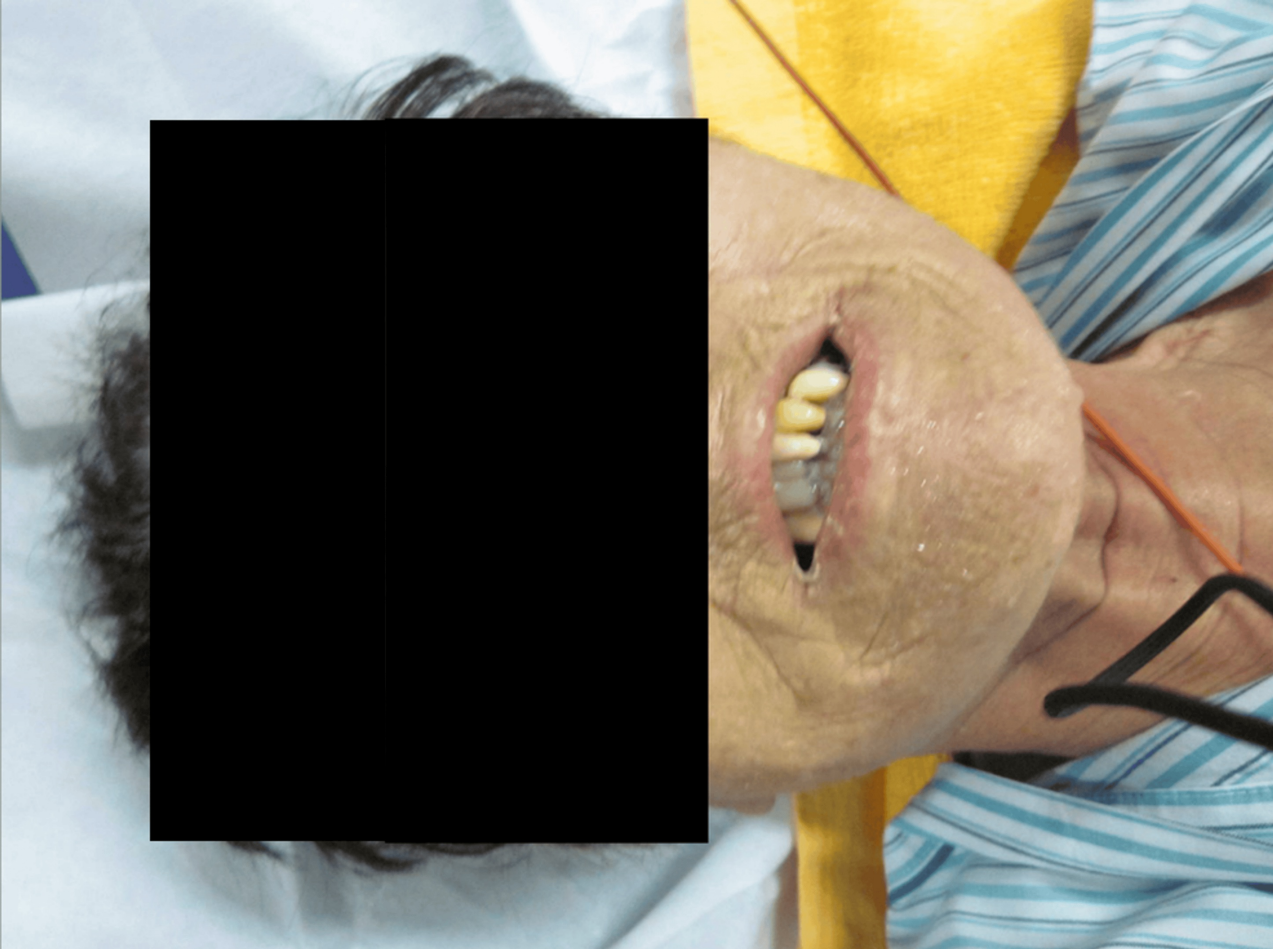
Limited mouth opening in the emergency department Marked sweating, limited mouth opening, and trismus are noted. These symptoms further supported the diagnosis of tetanus.

She visited a local clinic on Day -5, but the wound was not debrided, and she was discharged without receiving a tetanus toxoid vaccination. On Day -1, she presented to a general hospital due to pain and swelling in her right forearm. Hospitalization was recommended, but she refused. She received intravenous ceftriaxone and a tetanus toxoid vaccination as an outpatient before returning home. After returning home, she developed limited mouth opening, difficulty swallowing, excessive sweating, and limited extension in both elbows. On Day 1, due to worsening symptoms, she visited a general hospital again, where she was diagnosed with suspected tetanus and was subsequently referred to our hospital's emergency department on the same day. Physical examination revealed complete trismus (mouth opening: 0 cm), neck stiffness, and limited extension of the right elbow; the patient was diagnosed with Ablett Grade III (Figures 2, 3) [8].

**Figure 4:**
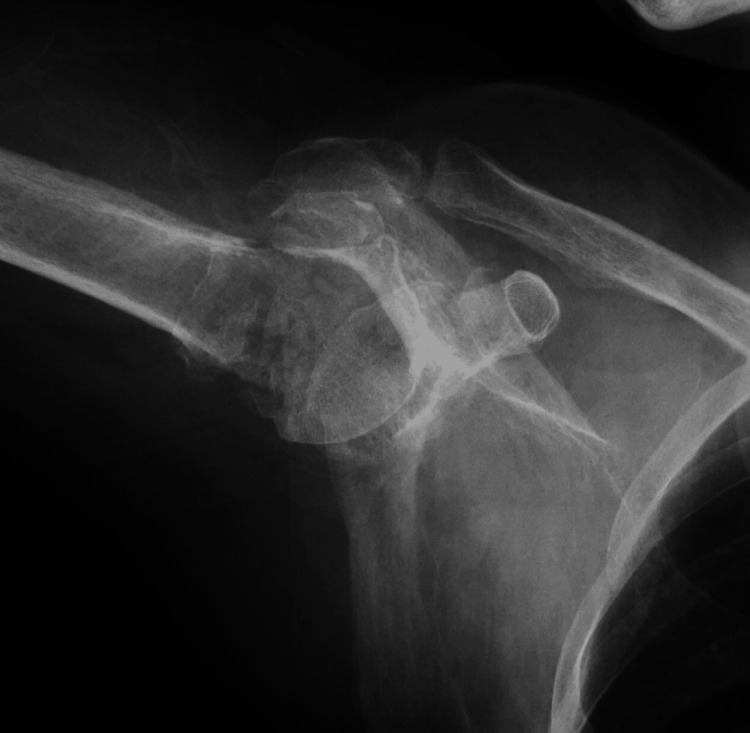
Plain radiograph on admission showing multiple fractures of the right upper extremity Right shoulder fracture-dislocation, proximal right humeral fracture, and right clavicle fracture are identified.

Due to persistent generalized muscle rigidity and frequent tonic spasms following the injury, concurrent right shoulder fracture-dislocation, proximal right humeral fracture, and right clavicle fracture were identified on Day 1 (Figure 4).

Vital signs on admission were blood pressure 196/121 mmHg, pulse 145 beats per minute (bpm), respiratory rate 24 breaths per minute, oxygen saturation (SpO₂) 99% on room air, and body temperature 36.6°C. The patient was admitted to the ICU for endotracheal intubation and mechanical ventilation [9]. The elevated white blood cell count on admission (174 × 10³/μL) was considered to reflect a systemic inflammatory response associated with tetanus infection and muscle injury. After ICU admission, autonomic storm and muscle rigidity persisted. To minimize external stimuli, the ICU room was kept dark, and the patient was provided with an eye mask and earplugs. Medications included sedatives and anticonvulsants (fentanyl, midazolam, and diazepam), a muscle relaxant (rocuronium), and anti-tetanus human immunoglobulin (TIG). Antibiotics (ceftriaxone, penicillin, and clindamycin), magnesium sulfate, and diltiazem were administered to manage autonomic symptoms [10].

The fractures were managed as follows. Right-sided fractures, identified on Day 1, were initially managed with skeletal traction to prevent redisplacement caused by tonic spasms during the acute phase. The proximal left humeral fracture, identified on Day 22, was managed conservatively. Conservative management for all fractures was agreed upon in discussion with the patient and her family. These fractures precluded active use of both upper limbs throughout the hospitalization, necessitating careful consideration of rehabilitation interventions and assistance methods to avoid excessive mechanical stress on the affected limbs. Subsequently, autonomic symptoms accompanied by opisthotonos and rapid fluctuations in vital signs appeared, and the condition progressed to Grade IV [8]. During this period, rehabilitation was not initiated, as the risk of exacerbating autonomic symptoms was considered high. Furthermore, tonic spasms were induced during positional changes, and the attending physician instructed staff to minimize physical stimulation as much as possible. Therefore, formal neurological assessment could not be safely performed.

Rehabilitation intervention phase (days 11-41)

**Figure 5:**
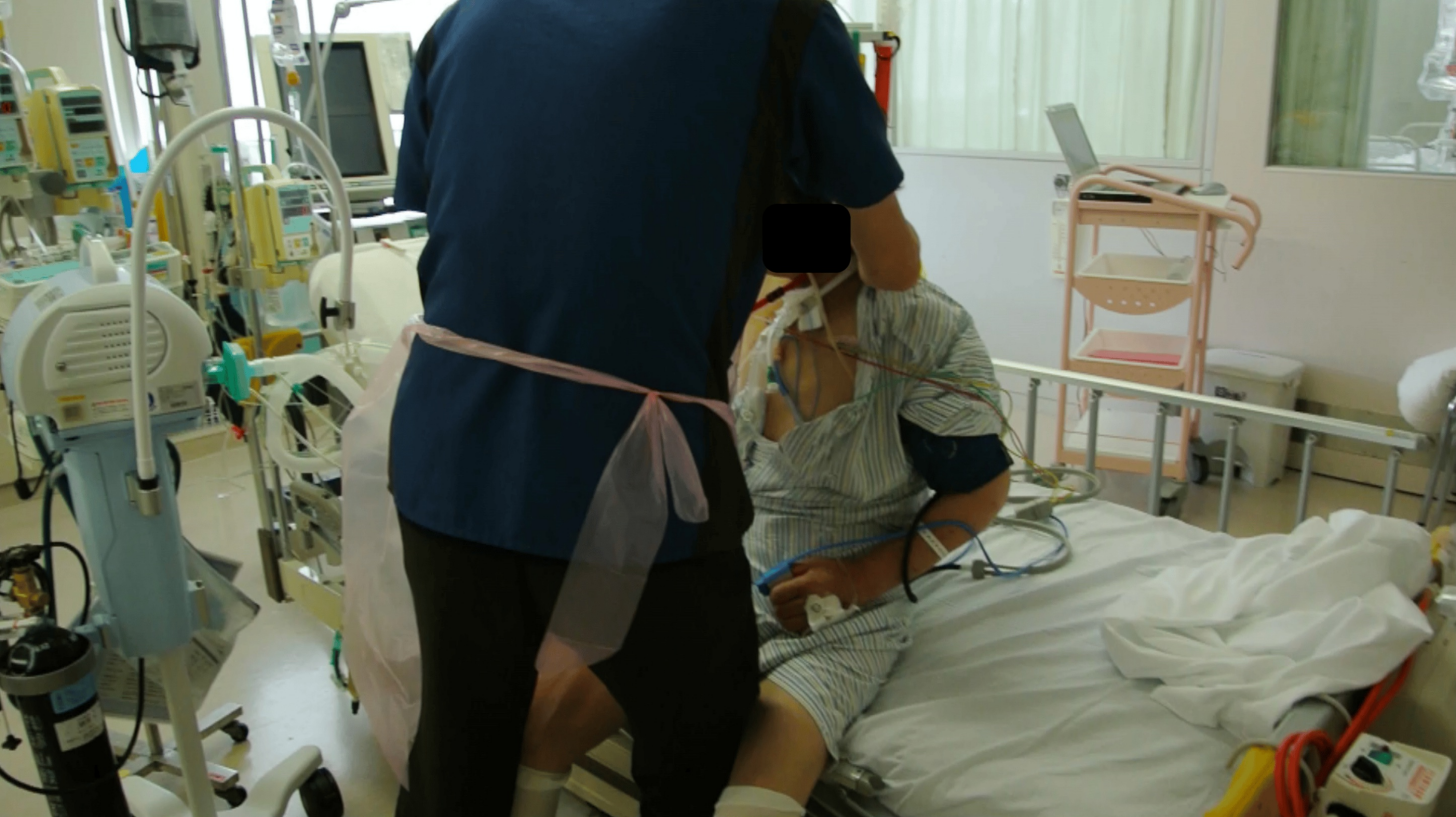
Rehabilitation session during the ICU stay The patient is shown performing edge-of-bed sitting while receiving mechanical ventilation. ICU: Intensive care unit.

Rehabilitation sessions were conducted twice daily, in the morning and afternoon, six days per week. Each session lasted approximately 20-40 minutes. The intensity was gradually adjusted according to the patient's physiological condition and tolerance at each stage of rehabilitation. On Day 11, resolution of muscle hypertonicity and opisthotonos was observed, along with a downward trend in CK levels and the stabilization of blood pressure and heart rate. Based on these clinical findings, passive range-of-motion exercises and respiratory physical therapy were initiated, while the patient continued to wear an eye mask and earplugs to minimize stimulation [6]. On Day 13, postural drainage and respiratory physical therapy were performed to improve pulmonary function on the dependent side. On Day 19, following a reduction in sedative dosage, sedation level was monitored using the Richmond Agitation-Sedation Scale (RASS) [11], and gradual responses to verbal cues and tactile stimulation were observed. The RASS was used with permission from Virginia Commonwealth University, Richmond, VA (© 2012, all rights reserved). On Day 22, plain radiographs revealed a fracture of the proximal left humerus, which was attributed to recurrent muscle hypertonicity and tonic spasms since admission. On Day 25, a tracheostomy was performed. By Day 26, the patient's level of consciousness had improved, and responses to verbal stimuli were observed. On Day 30, simple communication became possible. On Day 35, edge-of-bed sitting was initiated, and no apparent worsening of autonomic symptoms was observed (Figure 5).

From Day 35 onward, symptoms of delirium appeared, including disorientation and impaired comprehension. Delirium was assessed using the Confusion Assessment Method for the Intensive Care Unit (CAM-ICU) [12], and these symptoms were considered potentially consistent with cognitive dysfunction associated with PICS. The CAM-ICU was used in accordance with the open-access policy of the copyright holders (© 2002, E. Wesley Ely, MD, MPH, and Vanderbilt University, Nashville, TN, all rights reserved). On Day 41, mechanical ventilation was discontinued.

Mobilization phase (days 42-69)

On Day 42, wheelchair transfers were initiated. Mobilization was progressively advanced, and no apparent worsening of autonomic symptoms was observed throughout this phase [13]. Rehabilitation continued using a multidisciplinary approach involving occupational therapists, speech-language therapists, nurses, and others to address delirium [6]. On Day 53, improvements in joint range of motion and muscle strength were noted, and standing exercises were initiated [14].

Gait training and discharge (days 70-114)

Given the bilateral upper limb fractures, conventional assistive devices such as parallel bars or walkers could not be utilized. Therefore, standing exercises were performed with manual assistance to train antigravity muscles, and gait training was conducted with the therapist providing trunk support from behind, allowing safe progression of ambulation without upper limb loading. Gait training began on Day 70. The patient was unstable due to a posteriorly displaced center of gravity and reduced lower-leg muscle strength, requiring assistance. By Day 83, prolonged standing duration and increased walking distance were observed. On the same day, a multidisciplinary conference was held, attended by physicians, nurses, rehabilitation staff, and a medical social worker. During the conference, the patient and family expressed their wish to be discharged home, and home discharge was set as the goal. Guidance on caregiving methods was provided while gathering information about the home environment from the family. The home environment was assessed, including steps and daily living routes, and the introduction of long-term care insurance services was considered. By Day 86, the patient was able to walk with a quad cane with minimal assistance. On Day 88, walking distance increased to 50 m. On Day 92, as part of functional training focused on real-life activities, step negotiation exercises adapted to the home environment were conducted. On Day 113, the patient fell during a toilet transfer without nursing supervision; consequently, nursing supervision was initiated, and the level of assistance was increased. The patient was discharged home on Day 114. Throughout this phase, no apparent recurrence of autonomic symptoms was observed.

At discharge, basic mobility and walking were at a supervision level. Muscle strength of the lower limbs was assessed using the Medical Research Council (MRC) scale [15], with a total score of 18 (lower limb assessment only; maximum possible score: 30 out of 60). While the upper limbs could not be assessed due to the fractures, the patient was able to self-feed using a spoon with the left hand. The BI score was 55.

## Discussion

The dilemma of systemic management and PICS in severe tetanus

In the treatment of tetanus, isolation from external stimuli and sedation are necessary to control muscle rigidity and autonomic storm [3]. However, prolonged sedation and bed rest are associated with an increased risk of PICS [4]. While the risk of PICS is widely recognized in ICU management, early rehabilitation intervention is particularly challenging in tetanus, as external stimuli may trigger autonomic symptoms [2]. In this case, findings potentially consistent with PICS were observed in two domains: physical dysfunction and cognitive dysfunction. Regarding physical function, muscle weakness persisted at the time of discharge, and the BI score was lower than before admission, suggesting a possible contribution of ICU-acquired weakness (ICU-AW) resulting from prolonged deep sedation and bed rest [14,16]. It should also be noted that the BI score at discharge (55 points) was lower than the peak value recorded during hospitalization (70 points on Day 92). This decline was attributable to a fall that occurred on Day 113 during an unsupervised toilet transfer, which necessitated an increase in the level of supervision and assistance for mobility-related activities. Regarding cognitive function, delirium was identified using the CAM-ICU following the initiation of edge-of-bed sitting, and impaired orientation and comprehension were observed. These findings were considered potentially consistent with PICS. In cases of tetanus, minimization of external stimuli and deep sedation are essential for treatment, which may further increase the risk of developing PICS. Therefore, early multidisciplinary intervention may help mitigate this risk in such patients.

Challenges in rehabilitation intervention and lack of established guidelines

In recent years, the potential efficacy of early rehabilitation for critically ill patients in the ICU has been reported [5,13]. However, in cases of tetanus, external stimuli may trigger or exacerbate autonomic symptoms, making rehabilitation interventions potentially hazardous [6]. Consequently, clear guidelines regarding the timing of rehabilitation initiation and intervention intensity have not yet been established, and clinical practice relies on individualized clinical judgment [17].

Approach in this case: determining the intervention based on physiological indicators

In this case, criteria for initiating and continuing rehabilitation were established based on physiological indicators. The decision to begin intervention was made once all three of the following conditions were met: resolution of muscle hypertonicity and opisthotonos, a downward trend in CK levels, and stabilization of blood pressure and heart rate. These findings were considered to suggest the resolution of muscle hypertonicity and convulsions, reduction of muscle damage, and stabilization of hemodynamic parameters. In particular, the downward trend in CK levels was considered alongside other clinical findings as a potential indicator that excessive neural activity capable of triggering an autonomic storm may have subsided, though this interpretation remains speculative. By initiating intervention only after these conditions were met, rehabilitation was able to proceed without apparent recurrence of autonomic storm. Furthermore, excessively early intervention carries the risk of triggering a recurrence of autonomic symptoms, whereas delaying intervention may contribute to ICU-AW or functional decline [16]. Therefore, indicator-guided timing adjustment may be considered important for balancing these competing risks. However, it cannot be ruled out that the favorable outcome in this case was influenced by pharmacotherapy or the natural disease course, nor can it be definitively concluded that these indicators alone determined the outcome.

Unique characteristics of this case involving multiple fractures

In this case, persistent muscle rigidity had placed excessive mechanical stress on the bones and joints, and a right shoulder fracture-dislocation, a proximal right humeral fracture, and a right clavicle fracture were identified on admission. Additionally, a fracture of the left humerus was confirmed during hospitalization. Under these circumstances, early aggressive mobilization carries a potential risk of fracture displacement or additional injury. On the other hand, prolonged bed rest may contribute to joint contractures and disuse syndrome. Taking these competing risks into account, a stepwise rehabilitation approach with gradual load progression was implemented while minimizing external forces. In cases involving fractures complicated by muscle hypertonicity, individual adjustment of rehabilitation progression and assistance methods may be important. Compared with a previously reported case of tetanus with severe complications [6], the present case was distinctive in that bilateral humeral fractures necessitated modification of standard rehabilitation approaches, including the substitution of conventional assistive devices with manual trunk support during gait training.

The significance of stepwise rehabilitation

During rehabilitation, passive range-of-motion exercises and respiratory physical therapy were performed. Subsequently, the patient progressed stepwise through edge-of-bed sitting, wheelchair use training, standing practice, and walking practice. This stepwise approach allowed for gradual load progression while avoiding excessive external stimuli. Throughout the rehabilitation process, improvements in ADL were achieved without apparent exacerbation of autonomic symptoms. While such a stepwise approach is generally recommended for ICU rehabilitation [18], cases of tetanus may require particularly careful workload adjustment in consideration of external stimuli [6].

Multidisciplinary collaboration and family support

During hospitalization, physicians provided sedation and circulatory management; nurses managed stimulus control and positioning; and rehabilitation staff, including physical therapists (PT), occupational therapists (OT), and speech-language therapists (ST), supported functional recovery, provided caregiving guidance to the family, and conducted a home environment assessment. As the patient did not achieve full independence in ADL, discharge planning was led primarily by a medical social worker. To facilitate a return home, home-based rehabilitation and home nursing services were initiated, and the home environment was modified to establish a continuous support system. A multidisciplinary team was involved throughout the entire clinical course, from autonomic storm management in the ICU to rehabilitation interventions and discharge support. Consequently, the patient was discharged home in accordance with the wishes of the patient and family. These findings suggest that consistent multidisciplinary collaboration from the acute phase through the post-discharge period may contribute to functional recovery and return to home in cases of severe tetanus [19].

Limitations

This report describes a single case, and the generalizability of the findings is inherently limited. The favorable outcome observed in this case may have been influenced by pharmacotherapy, the natural course of the disease, or other confounding factors beyond the rehabilitation intervention itself. Furthermore, the physiological indicators used to guide the intervention were based on clinical judgment at a single institution; their objective validity, reproducibility, and applicability to other clinical settings require further verification. The relative contributions of each indicator also remain unclear, and it cannot be determined whether all three conditions were necessary or whether a subset would have been sufficient. In addition, the upper limb fractures prevented accurate objective assessment, including evaluation using the MRC scale [15], which represents a further limitation of this report. Additional case accumulation and, if possible, prospective evaluation will be necessary to verify the validity of the indicators and intervention strategies described here. The incorporation of other functional outcome measures in future cases would enable more accurate assessment.

## Conclusions

In this case, rehabilitation was performed in a patient with severe tetanus complicated by multiple fractures, resulting in successful discharge home. The decision to initiate rehabilitation was based on physiological indicators, including the resolution of muscle hypertonicity and opisthotonos, a downward trend in CK levels, and the stabilization of hemodynamic parameters. Based on these findings, rehabilitation proceeded without any apparent recurrence of autonomic storm during the intervention. The findings from this case may provide a basis for determining the timing of safe rehabilitation interventions in patients with severe tetanus. Further prospective studies with larger patient populations are needed to validate the physiological indicators and rehabilitation strategies described in this report. Multidisciplinary collaboration and individualized rehabilitation planning are considered essential for optimizing outcomes in similar cases.
